# Brachial artery reactivity in patients with severe sepsis: an observational study

**DOI:** 10.1186/cc11223

**Published:** 2012-03-05

**Authors:** Orren Wexler, Mary Anne M Morgan, Michael S Gough, Sherry D Steinmetz, Cynthia M Mack, Denise C Darling, Kathleen P Doolin, Michael J Apostolakos, Brian T Graves, Mark W Frampton, Xucai Chen, Anthony P Pietropaoli

**Affiliations:** 1 Division of Pulmonary and Critical Care Medicine, Department of Medicine, University of Rochester Medical Center, 601 Elmwood Avenue, Rochester, NY 14642, USA; 2 Echocardiography Laboratory, Division of Cardiology, Department of Medicine, University of Rochester Medical Center, 601 Elmwood Avenue, Rochester, NY 14642, USA; 3 Department of Nursing, University of Rochester Medical Center, 601 Elmwood Avenue, Rochester, NY 14642, USA; 4 Department of Respiratory Care, University of Rochester Medical Center, 601 Elmwood Avenue, Rochester, NY 14642, USA; 5 Department of Environmental Medicine, University of Rochester Medical Center, 601 Elmwood Avenue, Rochester, NY 14642, USA; 6 Center for Ultrasound Molecular Imaging and Therapeutics, Cardiovascular Institute, University of Pittsburgh Medical Center, 3550 Terrace Street, Pittsburgh, PA 15213, USA

## Abstract

**Introduction:**

Ultrasound measurements of brachial artery reactivity in response to stagnant ischemia provide estimates of microvascular function and conduit artery endothelial function. We hypothesized that brachial artery reactivity would independently predict severe sepsis and severe sepsis mortality.

**Methods:**

This was a combined case-control and prospective cohort study. We measured brachial artery reactivity in 95 severe sepsis patients admitted to the medical and surgical intensive care units of an academic medical center and in 52 control subjects without acute illness. Measurements were compared in severe sepsis patients versus control subjects and in severe sepsis survivors versus nonsurvivors. Multivariable analyses were also conducted.

**Results:**

Hyperemic velocity (centimeters per cardiac cycle) and flow-mediated dilation (percentage) were significantly lower in severe sepsis patients versus control subjects (hyperemic velocity: severe sepsis = 34 (25 to 48) versus controls = 63 (52 to 81), *P *< 0.001; flow-mediated dilation: severe sepsis = 2.65 (0.81 to 4.79) versus controls = 4.11 (3.06 to 6.78), *P *< 0.001; values expressed as median (interquartile range)). Hyperemic velocity, but not flow-mediated dilation, was significantly lower in hospital nonsurvivors versus survivors (hyperemic velocity: nonsurvivors = 25 (16 to 28) versus survivors = 39 (30 to 50), *P *< 0.001; flow-mediated dilation: nonsurvivors = 1.90 (0.68 to 3.41) versus survivors = 2.96 (0.91 to 4.86), *P *= 0.12). Lower hyperemic velocity was independently associated with hospital mortality in multivariable analysis (odds ratio = 1.11 (95% confidence interval = 1.04 to 1.19) per 1 cm/cardiac cycle decrease in hyperemic velocity; *P *= 0.003).

**Conclusions:**

Brachial artery hyperemic blood velocity is a noninvasive index of microvascular function that independently predicts mortality in severe sepsis. In contrast, brachial artery flow-mediated dilation, reflecting conduit artery endothelial function, was not associated with mortality in our severe sepsis cohort. Brachial artery hyperemic velocity may be a useful measurement to identify patients who could benefit from novel therapies designed to reverse microvascular dysfunction in severe sepsis and to assess the physiologic efficacy of these treatments.

## Introduction

Severe sepsis is characterized by impaired microvascular blood flow [1]. Microvascular function can be noninvasively assessed by measuring reactive hyperemia (RH), the augmentation in limb blood flow occurring after a period of stagnant ischemia [2]. The physiological mechanisms responsible for RH include release of the endothelium-dependent vasodilators nitric oxide (NO) and prostacyclin, activation of ATP-dependent potassium channels in smooth muscle, and a myogenic response [2-7]. Hypercoagulability and augmented adhesion and aggregation of leukocytes and erythrocytes may also contribute to a blunted RH response. [8-11].

Ultrasound measurement of hyperemic velocity (HV), the maximal velocity of blood flow after cuff deflation, can be used to assess RH [12-17]. Previous studies have shown that other indices of RH are impaired in human sepsis [8-11,18-24]. Many of these studies also reported associations between indices of RH and adverse outcomes, but did not fully explore whether these associations were confounded by important variables like vasopressor use, blood pressure, and comorbid conditions.

Endothelial dysfunction probably plays a major pathogenetic role in sepsis [25]. Ultrasound measurement of flow-mediated dilation (FMD) of the brachial artery is commonly used as a noninvasive measure of conduit artery endothelial function [26]. Flow-mediated dilation is largely mediated by endothelial NO production [26,27]. Increasing evidence supports dysregulated and insufficient NO activity in patients with sepsis syndrome [28-30]. To our knowledge, only one published study has examined FMD in human sepsis [31]. No published studies compare the ability of FMD and RH to predict outcomes in severe sepsis patients.

Ultrasound measurements of brachial artery reactivity have recently been used to simultaneously assess conduit artery endothelial function (with FMD) and microvascular function (with HV) [12-16]. Hyperemic velocity generates the brachial artery shear stress that is responsible for FMD, so the two measurements are clearly linked [32]. Nevertheless, in some studies of simultaneous FMD and HV measurements, HV alone (not FMD) has been associated with systemic inflammation [15], cardiovascular risk factors [14], and cardiovascular events [12]. Moreover, such studies have shown that FMD and HV are at best weakly correlated [13,14], suggesting that they reflect different physiologic processes: FMD estimating conduit-artery endothelial function and HV estimating RH and microvascular function [15-17].

The aims of this study were therefore to determine whether these two brachial artery reactivity measurements are independently associated with severe sepsis and hospital mortality, and to determine which of them is most predictive of mortality. We hypothesized that both brachial artery reactivity measurements would be reduced in severe sepsis patients and independently associated with hospital mortality. Some of these results have been reported previously in abstract form [33].

## Materials and methods

### Study design

This was a combined prospective cohort study and case-control study. The study design, clinical characteristics, and outcomes of many of the study subjects have been reported previously [29,30]. In brief, consecutive patients meeting diagnostic criteria for severe sepsis or septic shock [34] (subsequently collectively termed severe sepsis) in the medical or surgical intensive care unit (ICU) of the University of Rochester Medical Center were eligible. Control subjects without acute illness were recruited from the local community, stratified by age and gender to approximate the sepsis cohort. Prospectively defined exclusion criteria are listed in Table 1. Patients were also not enrolled if study technicians were unavailable to perform the measurements. All subjects or their surrogates provided written informed consent, and the study protocol was approved by the University of Rochester Research Subjects Review Board.

**Table 1:** Exclusion criteria

Code status limitations precluding critical care management (for example, directives against use of mechanical ventilation or vasopressor agents)
Refusal of patient or designated surrogate decision-maker to provide written informed consent, or inability to obtain consent within 48 hours of diagnosis

Severe cardiomyopathy with left-ventricular ejection fraction < 30%^a^

Chronic dialysis-dependent renal failure^a^

History of solid organ or bone marrow/stem cell transplantation^a^

Preexisting advanced liver disease (Child-Pugh grade C)^a^

Organic nitrate therapy^a^

Current active bleeding^a^

Hematocrit < 22% or < 25% while taking vasopressors^a^

Pregnancy or hormone replacement therapy (HRT)^a^

More than 48 hours since severe sepsis/septic shock diagnosis

Vascular-access device present in the target upper extremity^b^

Absent Doppler signals in target upper extremity

Skin breakdown or soft tissue inflammation involving target upper extremity^a^

History of vascular or lymphatic surgery involving target upper extremity^a^

^a^Exclusion criteria for both control subjects and sepsis patients. Control subjects also were excluded if they had infections or used antibiotics within 6 weeks of specimen collection.

*^b^*This exclusion criterion was included because of theoretic concerns that the procedure inducing stagnant ischemia could disrupt or displace a vascular-access catheter.

### Brachial artery reactivity

Brachial artery reactivity was measured by registered sonographers according to published guidelines [26]. Measurements occurred as soon as possible after diagnosis in severe sepsis patients, but were delayed in some cases until the clinical team decided (for reasons unrelated to the conduct of the study) to remove vascular-access devices from the target upper extremity. Measurements were made in temperature-controlled rooms within the ICU (severe sepsis patients) or the Clinical Research Center (control subjects), and attempts were made to minimize noise and other distractions. Subjects were placed in the supine position with approximately 30 degrees head elevation for at least 10 minutes before measurements. Subjects were not fasting at the time of measurements. The brachial artery was imaged by using a medial approach 2 cm above the antecubital fossa with the arm extended and the thumb pointed to the ceiling. The pulse-wave Doppler gate was positioned at a 60-degree angle within the center of the arterial lumen. All images were acquired by using a General Electric Vivid 7 ultrasound machine with a M12L transducer at a frequency of 14 MHz (GE Medical Systems, Milwaukee, WI, USA) or a Siemens Sequoia 512 ultrasound machine with a 15L8 transducer at a frequency of 13 MHz (Siemens Medical Solutions, Mountain View, CA, USA).

A sphygmomanometric cuff was placed at the widest part of the forearm 1 to 2 cm distal to the antecubital fossa. Preocclusion two-dimensional (2-D) gray-scale images and pulse-wave spectral Doppler recordings were obtained. The cuff was rapidly inflated to 200 mm Hg (or 50 mm Hg above systolic blood pressure if systolic blood pressure was more than 150 mm Hg) for 5 minutes, and then rapidly and completely deflated. Pulse-wave spectral Doppler recordings were acquired for 15 seconds after cuff deflation. Two-dimensional images were obtained 30 to 90 seconds after deflation at approximately 15-second intervals. Multiple images at baseline and after occlusion of the 2-D and Doppler recordings were digitally stored for later analysis.

A single sonographer blinded to clinical details and patient outcomes performed all analyses. The brachial artery diameter was measured at end-diastole, determined by the R wave from a simultaneously recorded telemetry tracing. Diameters were measured with electronic calipers by using ultrasonically identified anatomic landmarks, ensuring a consistent measurement location before and after cuff occlusion in each subject. The diameter of the brachial artery was measured from the media-adventitia interface in the near field to the media-adventitia interface in the far field. A series of three diameter measurements was averaged at baseline and after deflation. The three maximal postdeflation diameter measurements were used. The percentage brachial artery FMD was calculated as the difference between brachial artery diameter after and before occlusion, divided by preocclusion brachial artery diameter. The velocity-time integral (VTI) over a single cardiac cycle was calculated from the pulse-wave spectral Doppler tracing (units = centimeters/cardiac cycle). The baseline velocity was considered the average of three representative Doppler tracings before brachial artery occlusion. Hyperemic velocity was considered the average of the three maximal Doppler tracings 0 to 15 seconds after cuff release.

To assess intraobserver variability in brachial artery diameter and velocity measurements, we reanalyzed the digital images from a consecutive sample of 22 studies 6 months or more after the original measurements in a blinded fashion.

### Statistical analysis

Results are expressed as mean ± standard deviation (SD) or median (interquartile range (IQR)), as appropriate. The Student *t *test, the Wilcoxon rank-sum test, or the Wilcoxon matched-pairs signed-ranks test was used to compare continuous or discrete variables. The χ^2 ^test or Fisher Exact test was used to compare categoric variables. The Spearman rank correlation coefficients (rho) were calculated between continuous variables. The Kaplan-Meier method was used to assess the relation between 6-month survival and brachial artery reactivity measurements [35].

The primary independent variables were brachial artery reactivity measurements. The primary outcome measures were severe sepsis (severe sepsis patients versus control subjects) and hospital mortality (survivors versus nonsurvivors). Secondary outcome variables included sequential organ-failure assessment (SOFA) scores, number of organ failure-free days from days 0 to 28, number of ventilator-free days from days 0 to 28, number of ICU-free days from days 0 to 28, and 6-month survival after diagnosis [36-38].

A medical history of hypertension, hyperlipidemia, smoking, diabetes mellitus, coronary artery disease, age, gender, mean arterial pressure, and the Charlson comorbidity index were considered potentially important covariables that could be associated with brachial artery reactivity and severe sepsis [29,39-41]. These covariables and the presence or absence of vasopressor infusions (at the time of brachial artery measurements) were considered potentially important covariables that could be associated with brachial artery reactivity and hospital mortality. We therefore conducted stratified analyses to determine whether any of these individual covariables confounded observed associations between brachial artery reactivity and severe sepsis or hospital mortality. We also used these stratified analyses to look for interactions between these covariables and brachial artery reactivity in predicting the primary outcomes (severe sepsis and hospital mortality) [42].

We next performed multivariable logistic regression analyses by using the aforementioned covariables to determine the independent association between brachial artery reactivity and the primary outcomes. Covariables least associated with the outcome of interest were sequentially removed from the logistic regression models if the likelihood ratio test comparing nested models remained insignificant (*P *> 0.10), until additional variables could not be removed. The brachial artery reactivity measurements were then introduced into the parsimonious model. Logistic regression model performance was assessed by using the C statistic and Hosmer-Lemeshow test [43].

The receiver-operating characteristic (ROC) curves for brachial artery reactivity measurements were compared [44], and the sensitivity and specificity of the optimal values were calculated. Measurement error between the two paired measurements was assessed by using the methods of Bland and Altman [45], and the kappa statistic was used to assess their level of agreement in classifying patients [46]. Statistical significance was accepted at *P *< 0.05. Statistical analyses were performed by using SAS 9.2 and Stata 9.1.

## Results

**Figure 1:**
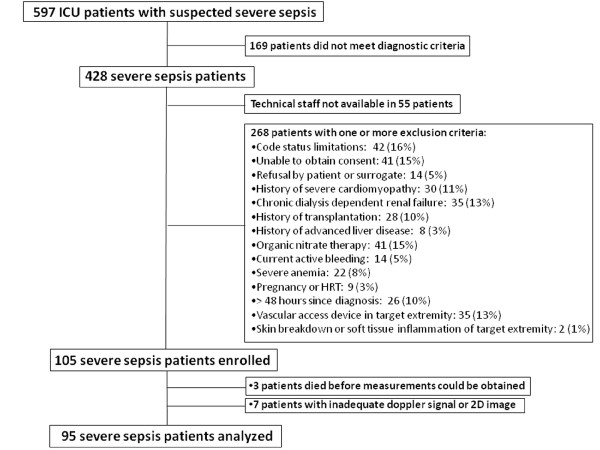
**Enrollment algorithm for severe sepsis patients**.

Between February 2006 and February 2009, 102 severe sepsis subjects were enrolled, and 95 had adequate brachial artery reactivity measurements (Figure 1). Seven enrolled patients were not analyzed because 2-D images were inadequate (*n *= 4), Doppler images were inadequate (*n *= 2), or images were lost (*n *= 1). Fifty-two control subjects without acute illness were recruited. In general, the procedure was well tolerated. Several subjects noted mild discomfort during the stagnant forearm ischemia that rapidly resolved after cuff deflation. No adverse events occurred.

Clinical characteristics of the study subjects are shown in Table 2. Brachial artery reactivity was measured 41 (30 to 57) hours after patients met severe sepsis diagnostic criteria. As shown in Table 2, 85% of our severe sepsis patients were in septic shock at the time of diagnosis. However, most patients had recovered normal blood pressure (mean arterial pressure = 80 (72 to 90) mm Hg), and only 28% required vasopressor infusions when brachial artery reactivity was measured (Table 2), indicating some degree of cardiovascular stabilization by the time measurements were performed.

**Table 2:** Clinical characteristics of study subjects^*a*^

	Controls	Severe sepsis	*p*	Survivors	Nonsurvivors	*P*
	(*n *= 52)	(*n *= 95)		(*n *= 78)	(*n *= 17)	
Age (years)	60 (53-66)	62 (49-74)	0.42	58 (48-71)	74 (64-78)	0.006
Male gender	26 (50%)	49 (52%)	0.85	43 (55%)	6 (35%)	0.14
Race			0.36^*c*^			0.37
Caucasian	49 (94%)	80 (84%)	-	67 (86%)	13 (76%)	-
African-American	3 (6%)	12 (13%)	-	9 (12%)	3 (18%)	-
Asian	0	1 (1%)	-	1 (1%)	0 (0)	-
Hispanic/Latino	0	2 (2%)	-	1 (1%)	1 (5%)	-
Hypertension history	11 (21%)	59 (62%)	< 0.001	44 (56%)	15 (88%)	0.01
Hyperlipidemia	16 (31%)	34 (36%)	0.54	26 (33%)	8 (47%)	0.28
Current tobacco use	1 (2%)	23 (24%)	< 0.001	20 (26%)	3 (18%)	0.76
MAP (mm Hg)	91 (84-100)	80 (72-90)	< 0.001	82 (73-91)	79 (71-87)	0.31
Heart rate (beats/min)	61 (54-68)	87 (77-98)	< 0.001	86 (77-98)	87 (80-102)	0.58
Temp (°C)	-	36.9 ± 1.0	-	37.0 ± 1.01	36.9 ± 0.78	0.79
Charlson index	0 (0-1)	3 (1-5)	< 0.001	2.5 (1-5)	4 (2-8)	0.05
Medical patient	-	85 (89%)	-	74 (95%)	11 (65%)	0.002
Surgical patient	-	10 (11%)	-	4 (5%)	6 (35%)	
Site of infection			-			0.47
Pulmonary	-	57 (60%)	-	45 (58%)	12 (70%)	--
Intraabdominal	-	11 (12%)	-	9 (12%)	2 (12%)	--
Urinary	-	11 (12%)	-	11 (14%)	0	--
Skin/catheter	-	4 (4%)	-	4 (5%)	0	--
Other	-	12 (13%)	-	9 (12%)	3 (18%)	--
Microbiology						0.63
Gram-^+ ^bacteria	-	30 (32%)	-	23 (29%)	7 (41%)	--
Gram-^- ^bacteria	-	16 (17%)	-	13 (17%)	3 (18%)	--
Fungal	-	3 (3%)	-	3 (3%)	0	--
Mixed or other	-	17 (18%)	-	13 (17%)	4 (24%)	--
Unknown	-	29 (30%)	-	26 (33%)	3 (18%)	--
Positive blood culture	-	33 (35%)	-	27 (35%)	6 (35%)	0.96
Vasopressor use^b^	-	27 (28%)	-	19 (24%)	8 (47%)	0.08
Septic shock^c^	-	73 (85%)	-	58 (83%)	15 (94%)	0.45
APACHE II score	-	23 ± 8	-	21.8 ± 8.0	28.3 ± 7.2	0.003
Dysfunctional organs^d^						0.04
1	-	14 (15%)	-	14 (18%)	0	--
2	-	34 (36%)	-	30 (38%)	4 (24%)	--
3	-	26 (27%)	-	20 (26%)	6 (35%)	--
≥ 4	-	21 (22%)	-	14 (18%)	7 (41%)	--

Charlson index, Charlson comorbidity index [40]; MAP, mean arterial pressure at the time of brachial artery reactivity measurements; Temp, temperature at the time of brachial artery reactivity measurements; APACHE II, acute physiology and chronic health evaluation II [37]; ^a^Values are median (interquartile range), number (percentage), or mean (± SD); ^b^vasopressor use, continuous intravenous infusion of one or more vasopressor agents (norepinephrine, phenylephrine, epinephrine, dopamine, vasopressin) coincident with measurements of brachial artery reactivity; ^c^shock, hypotension or vasopressor dependence that persisted for ≥ 3 hours despite fluid challenge at the time of diagnosis; ^d^organ dysfunctions as defined previously [65] with slight modification: cardiovascular (hypotension (systolic blood pressure < 90 mm Hg or MAP < 60 mm Hg), vasopressor requirement, or clinical evidence of hypoperfusion); acid-base (metabolic acidosis and plasma lactate concentration > 2 m*M*); renal (urine output < 0.5 ml/kg/h despite fluid resuscitation); neurologic (altered mental status without other causes); respiratory (P:F ratio < 250, or < 200 if lungs are the only dysfunctional organs); hematologic (platelet count < 80,000 or > 50% decrease from baseline)

### Severe sepsis versus control subjects

**Figure 2:**
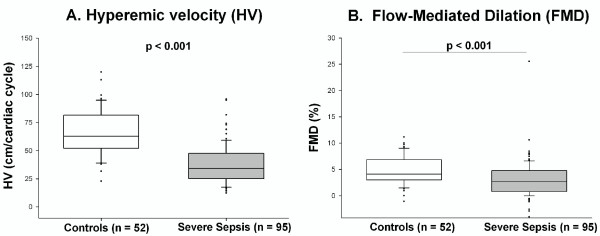
**Brachial artery reactivity in severe sepsis patients versus control subjects: hyperemic velocity (a) and flow-mediated dilation (b)**. Box plots show the median (horizontal line), 25^th^, and 75^th ^percentiles (lower and upper limits of the box). The dots represent outliers beyond the whiskers that designate the 10^th ^and 90^th ^percentiles. Comparisons made with the Wilcoxon rank-sum test.

Compared with control subjects, severe sepsis patients had significantly lower FMD (controls = 4.11 (3.06 to 6.78)%, severe sepsis = 2.65 (0.81 to 4.79)%; *P *< 0.001) and HV (controls = 63 (52 to 81) cm/cardiac cycle, severe sepsis = 34 (25 to 48) cm/cardiac cycle; *P *< 0.001; Table 3 Figure 2). Lower HV in severe sepsis versus control subjects was not explained by differences in the duration of the cardiac cycle because the baseline velocity-time integral was similar in the two groups (Table 3).

**Table 3:** Brachial artery reactivity measurements

	Control	Severe sepsis	*P *value	Survivors (*n *= 78)	Nonsurvivors (*n *= 17)	*P *value
Diameter (cm) before	0.40 (0.35-0.47)	0.43 (0.36-0.50)	0.15	0.44 (0.37-0.50)	0.38 (0.33-0.49)	0.24
Diameter (cm) after	0.42 (0.37-0.49)	0.44 (0.37-0.52)	0.31	0.45 (0.39-0.52)	0.40 (0.34-0.49)	0.15
FMD (%)	4.11 (3.06-6.78)	2.65 (0.81-4.79)	< 0.001	2.96 (0.91-4.86)	1.90 (0.68-3.41)	0.12
Baseline velocity (cm/cardiac cycle)	10 (7-14)	11 (8-15)	0.71	11 (8-16)	8 (7-12)	0.06
Hyperemic velocity (cm/cardiac cycle)	63 (52-81)	34 (25-48)	< 0.001	39 (30-50)	25 (16-28)	< 0.001
Change in velocity (cm/cardiac cycle)	54 (39-69)	23 (15-32)	< 0.001	25 (18-38)	13 (8-15)	< 0.001

FMD, flow-mediated dilation; HV, hyperemic velocity; diameter, brachial artery diameter.

Stratified analyses showed that the association between severe sepsis and FMD depends on age category. For subjects younger than 60 years (the median age of cases and controls combined), FMD was lower in severe sepsis patients (2.65 (0.91 to 4.13)%; *n *= 45) than control subjects (4.82 (3.76 to 8.33)%; *n *= 30; *P *< 0.001). For subjects older than 60 years, FMD was similar in severe sepsis (2.80 (0.77 to 5.31)%; *n *= 50) and control subjects (3.56 (1.80 to 5.92)%; *n *= 22; *P *= 0.34). The test for interaction was significant (*P *< 0.02), confirming that the relation between FMD and severe sepsis depended on age category. Among the other covariables, no confounding or effect modification was identified, although only one smoking control subject was tested, so tobacco use could not be fully evaluated (for complete results of this stratified analyses, see Tables E1 to E2 of Additional file 1 online data supplement).

Moderate correlation was found between FMD and HV in the combined study sample (Spearman rho = 0.38; *P *< 0.001; *n *= 147). This was primarily accounted for by the control subjects (Spearman rho = 0.44; *P *= 0.001; *n *= 52) because the correlation in severe sepsis subjects alone was poor (Spearman rho = 0.18; *P *= 0.08; *n *= 95).

Multivariable analyses assessing the independent relations between brachial artery reactivity and severe sepsis began with all specified covariables except smoking. The final logistic regression model included age, gender, history of hypertension, mean arterial pressure at the time of measurements, and Charlson comorbidity index. This model had excellent discrimination (C statistic = 0.91) but poor calibration (Hosmer-Lemeshow χ^2 ^= 119; *P *< 0.001). We performed two multivariable analyses to assess the independent relation between FMD and severe sepsis because of the aforementioned age-FMD interaction. In subjects 60 years or younger, lower FMD was independently associated with severe sepsis (odds ratio (OR) for severe sepsis per 1% decrease in FMD = 1.64; 95% confidence interval (CI) = 1.15 to 2.35; *P *< 0.01). In contrast, FMD was not independently associated with severe sepsis in subjects older than 60 years (OR for severe sepsis per 1% decrease in FMD = 1.07, 95% CI = 0.90 to 1.26; *P *= 0.45). Hyperemic velocity was independently associated with sepsis in the multivariable model (OR for severe sepsis per 1 cm/cardiac-cycle decrease in HV = 1.05; 95% CI = 1.02 to 1.08; *P *= 0.001). For complete results of these multivariable analyses, see Tables E3 and E4 of Additional file 1 online data supplement.

### Relation of brachial artery reactivity to outcomes and severity of illness in severe sepsis

**Figure 3:**
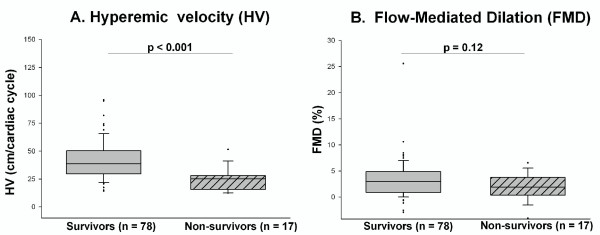
**Brachial artery reactivity in severe sepsis survivors versus nonsurvivors: hyperemic velocity (a) and flow-mediated dilation (b)**. Box plots show the median (horizontal line), 25^th^, and 75^th ^percentiles (lower and upper limits of the box). The dots represent outliers beyond the whiskers that designate the 10^th ^and 90^th ^percentiles. Comparisons made with the Wilcoxon rank-sum test.

Seventeen of the enrolled severe sepsis patients died before hospital discharge, 14 of the original sepsis episode, one during a subsequent sepsis episode, and two of stroke after sepsis resolution. FMD tended to be lower in nonsurvivors, but the difference was not statistically significant (survivors = 2.96 (0.91 to 4.86)%; nonsurvivors = 1.90 (0.68 to 3.41)%; *P *= 0.12; Figure 3, Table 3). In contrast, HV was significantly lower in nonsurvivors (survivors = 39 (30 to 50) cm/cardiac cycle; nonsurvivors = 25 (16 to 28) cm/cardiac cycle; *P *< 0.001; Figure 3, Table 3). The change in velocity (HV minus baseline velocity) was also significantly lower in nonsurvivors versus survivors (Table 3), indicating that the lower HV in nonsurvivors was indeed reflecting lower RH (and not simply a reflection of the marginally lower baseline velocity). In stratified analysis, HV remained lower in nonsurvivors within subgroups of all prespecified covariables (Table 4). The time interval from sepsis diagnosis to brachial artery measurements was similar in survivors and nonsurvivors (survivors = 41 (29 to 56) hours; nonsurvivors = 44 (36 to 71) hours; *P *= 0.26).

**Table 4:** Hyperemic velocity in survivors versus nonsurvivors: stratified analysis^a^

**Covariable**	**Survivors**	**Nonsurvivors**	***P* ^b^**
			
Age			
≤ 62	38 (29-52, *n *= 45	16 (14-22), *n *= 4	0.005
> 62	39 (32-48), *n *= 33	25 (21-28), *n *= 13	0.003
Gender			
Men	37 (29-46), *n *= 43	19 (15-22), *n *= 6	0.001
Women	42 (30-52), *n *= 35	26 (17-28), *n *= 11	0.009
Hypertension			
Yes	38 (27-49), *n *= 44	25 (16-28), *n *= 15	0.001
No	39 (30-51), *n *= 34	17 (12-21), *n *= 2	0.027
Diabetes mellitus			
Yes	38 (26-53), *n *= 18	17 (15-22), *n *= 9	< 0.001
No	39 (30-48), *n *= 60	28 (26-34), *n *= 8	0.054^*c*^
Hyperlipidemia			
Yes	36 (26-46), *n *= 26	22 (16-28), *n *= 8	0.024
No	39 (30-51), *n *= 52	25 (16-27), *n *= 9	< 0.001
Active smoking			
Yes	38 (30-53), *n *= 20	16 (12-28), *n *= 3	0.022
No	39 (30-48), *n *= 58	25 (17-28), *n *= 14	< 0.001
Coronary artery disease			
Yes	39 (32-46), *n *= 13	19 (14-24), *n *= 4	0.007
No	38 (29-51), *n *= 65	25 (17-28), *n *= 25	0.001
Pressors			
Yes	36 (25-44), *n *= 19	19 (13-28), *n *= 8	0.03
No	39 (30-51), *n *= 59	25 (21-28), *n *= 9	0.001
**Blood pressure**			
MAP ≤ 80 mm Hg	37 (29-46), *n *= 37	17 (14-27), *n *= 11	0.001
MAP > 80 mm Hg	41 (30-51), *n *= 41	26 (22-29), *n *= 6	0.018
**Charlson index**			
≤ 3	37 (29-51), *n *= 47	25 (17-34), *n *= 8	0.020
> 3	42 (30-48), *n *= 31	22 (15-27), *n *= 9	0.001

MAP, mean arterial pressure at the time of brachial artery reactivity measurements; n/a, not applicable. ^a^Continuous variables were dichotomized according to their median value (in the severe sepsis patients alone) for this analysis. ^b^*P *value refers to the comparison of HV between survivors and nonsurvivors within the specified subgroup. The borderline statistical significance in this group suggested an interaction between diabetes mellitus and HV in predicting hospital mortality was possible (see Methods). However, the test for interaction [42] was insignificant (*P *> 0.20).

In multivariable analysis beginning with all of the specified covariables, the final model included age and medical history of diabetes mellitus. This model had very good discrimination and calibration (C statistic = 0.77; H-L χ^2 ^= 5.2; *P *= 0.78). When controlling for these covariables, HV was an independent predictor of hospital mortality: the odds ratio for hospital mortality per 1-cm/cardiac cycle decrease in HV was 1.11 (95% CI = 1.04 to 1.19; *P *= 0.003; see Table E5 of Additional file 1 online data supplement).

### Secondary outcome measures

**Figure 4:**
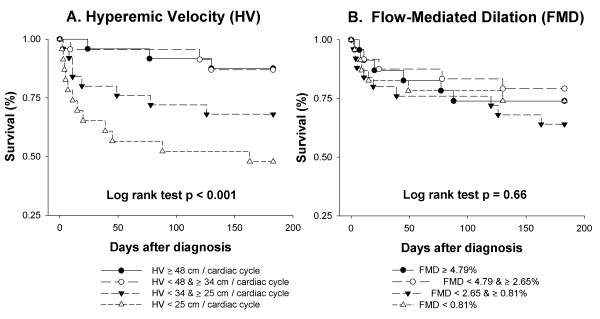
**Kaplan-Meier survival probability plots for quartiles of hyperemic velocity (a) and flow-mediated dilation (b)**. No subjects were lost to follow-up. The log-rank test was used to evaluate the statistical significance of the trend in survival per quartile of brachial artery reactivity.

HV was significantly negatively correlated with maximum and median SOFA scores from days 0 through 7, and significantly positively correlated with the number of organ failure-, ICU-, and ventilator-free days from days 0 to 28 (Table 5). FMD was not correlated with any of these variables. Quartiles of HV, but not FMD, predicted survival over the 6 months after severe sepsis diagnosis (Figure 4).

**Table 5:** Correlations of brachial artery reactivity with severity of illness/secondary outcomes

	HV		FMD	
		
	Rho	*P *value	Rho	*P *value
SOFA (mean)	0.274	0.007	-0.102	0.326
SOFA (maximum)	0.262	0.010	-0.108	0.296
Organ failure-free days, days 0 to 28	0.339	< 0.001	0.050	0.630
ICU-free days, days 0 to 28	0.299	0.003	0.063	0.546
Ventilator-free days, days 0 to 28	0.336	< 0.001	0.189	0.066

HV, hyperemic velocity; FMD, flow-mediated dilation; SOFA (mean), mean sequential organ-failure assessment score (days 0 to 7 after diagnosis);

SOFA (maximum), maximal sequential organ-failure assessment score (days 0 to 7 after diagnosis).

### Receiver operator characteristics analysis

The area under the curve (AUC) was higher (*P *= 0.03) for HV (0.82; 95% CI = 0.71 to 0.93) than for FMD (0.62; 95% CI = 0.48 to 0.77). The optimal HV cut-point for predicting mortality was 29 cm/cardiac cycle, with sensitivity of 88% and specificity of 77%. The optimal FMD cut-point for predicting mortality was 1.98%, with a sensitivity of 59% and a specificity of 68%.

### Intraobserver variability

Repeated measurements performed by the same sonographer blinded to the first measurement were highly correlated (intraclass correlation coefficient was 0.80 for FMD and 0.97 for HV). However, the paired FMD measurements appeared to stray from the line of identity (see Figure E1 of Additional file 1 online data supplement). Intraobserver repeatability was assessed by using the methods of Bland and Altman [45] (see Figure E2 of Additional file 1 online data supplement). The coefficient of repeatability (the expected difference between repeated measurements for 95% of paired observations) was 4.1% for FMD and 10 cm/cardiac cycle for HV. Next, we assessed whether agreement existed between paired values when classifying subjects. Measurements were dichotomized into "normal" or "septic" categories based on the median values in control subjects. Excellent agreement was found between the paired values for HV (kappa = 0.89) but only fair agreement for FMD (kappa = 0.45). Further analysis indicated that the paired measurements of baseline and hyperemic brachial artery diameter were precise (intraclass correlation coefficients were 0.99, and the coefficients of repeatability were 0.02 cm for both baseline and hyperemic brachial artery diameter; see Figures E1 and E3 of Additional file 1 online data supplement).

This analysis indicates that although arterial diameter measurements were precise and repeatable, the repeatability of percent FMD was poor, even when performed by the same highly trained sonographer. As a result, FMD-based patient classification was prone to error. In contrast, HV was highly repeatable, and paired measurements had excellent agreement in classifying patients.

## Discussion

In this study of brachial artery reactivity in severe sepsis patients, our main findings were that lower HV was independently associated with severe sepsis and hospital mortality. Lower brachial artery HV was also associated with higher severity of illness and lower 6-month survival. In contrast, although FMD was lower in sepsis patients than in control subjects, it did not predict severity of illness or adverse outcome.

### Implications of low HV in severe sepsis

The physiologic mechanisms responsible for RH originate in the microvasculature and are both endothelium dependent and endothelium independent [3-7]. They include a direct physical myogenic response [6], opening of ATP-sensitive potassium channels [3], and production of vasodilator substances, including prostaglandins [6], nitric oxide [5], and adenosine [3-5,7]. Microvascular obstruction from microthrombi, leukocyte adhesion, or sepsis-associated erythrocyte dysfunction may also contribute to impaired RH in sepsis [8-11]. Our HV measurements do not allow us to decipher which of these physiological mechanisms are most disrupted or most lethal in severe sepsis. However, they do imply that dysregulation of one or more of these microvascular mechanisms, in concert, is responsible for the abnormal HV we observed in severe sepsis patients and nonsurvivors.

Our findings are consistent with previous studies showing that other indices of RH are reduced in sepsis [8-11,18,19], associated with illness severity [20-22], and associated with ICU mortality [23]. Our study adds to these reports by uniquely demonstrating that HV remains independently associated with hospital mortality when specifically controlling for comorbidity, vasopressor use, or blood pressure (Table 4), and also in multivariable analysis (Table 5).

The finding that HV independently predicts severe sepsis mortality supports the concept that microvascular dysfunction is a central pathophysiologic process responsible for organ dysfunction and poor outcomes in sepsis [25,47]. In this context, treatments designed to improve microvascular function and clinical outcomes should be evaluated. For example, previous studies suggest that antioxidants may have benefit in critical illness [36,48], and they improve indices of RH and reduce inflammatory markers in other patient groups [49]. It seems logical to investigate this same possibility in severe sepsis. In addition, NO donors improve microcirculatory flow in sepsis patients [50]. Current efforts are under way to harness the beneficial effects of NO without causing the hypotension induced by organic nitrates [25]. Indices of RH could serve as physiological biomarkers in trials of such agents, ensuring that appropriate severe sepsis patients with abnormal microvascular function are enrolled in studies designed to affect this mechanistic pathway. This type of study design is now recommended for clinical trials in critical care to overcome the patient heterogeneity that dilutes precise measurement of therapeutic effectiveness [51,52]. Along the same lines, indices of RH could be used to test whether the therapeutic intervention is having the intended physiologic effects and clinical benefits, thereby substantiating a clinically relevant mechanistic pathway.

### Previous measures of RH in sepsis

Several techniques have been used to assess RH and microvascular function in patients with severe sepsis. Previous studies of RH in sepsis by using plethysmography are limited by small sample size [9-11,53]. Nevertheless, because plethysmography was the primary noninvasive method for determining the physiological mechanisms of RH in humans, it most closely approaches a gold-standard noninvasive measurement technique. Therefore, if other noninvasive methods are being used to investigate RH in sepsis patients, they should closely correlate with plethysmography.

A previous report showed that skin blood flow after stagnant ischemia, estimated by using transcutaneous laser Doppler measurements of erythrocyte velocity, was reduced in sepsis [19]. However, a variable relation exists between the laser Doppler measurements and plethysmographic forearm blood-flow measurements, and these measurements vary with slight changes in skin-probe location [54].

Reactive hyperemia peripheral arterial tonometry (RH-PAT) volumetrically measures digital pulse-wave amplitude in response to stagnant ischemia [55]. Sepsis-associated reductions in RH-PAT that are correlated with severity of illness have been observed [20]. Advantages of RH-PAT are that the computer-generated results are user independent, minimal training is involved, and the results are repeatable. Disadvantages are that the relation between RH-PAT and plethysmography is unknown, and it requires specialized and costly equipment.

Near-infrared spectroscopy plethysmography (NIRS) measures the change in microvascular hemoglobin levels and oxygen saturation during RH [22]. Blood flow estimated by NIRS was tightly correlated with plethysmography in normal subjects at rest, although the correlation was weaker after exercise [56]. NIRS-derived tissue oxygen consumption and tissue reoxygenation rate (or slope) after stagnant ischemia have been associated with sepsis, severity of illness, and clinical outcomes [18,22-24]. Disadvantages of NIRS are that it requires specialized equipment and disposable yet costly probes, and tissue fat and edema can produce interference that can impair accuracy.

### Technical considerations of brachial artery reactivity measurement

Optimal comprehensive ultrasound measurement of brachial artery reactivity parameters (including FMD) requires extensive technical expertise, particularly the quantification of brachial artery diameter [26]. We therefore required that all our studies be performed by experienced, registered sonographers. This requirement often delayed our measurements and limits the widespread clinical application of comprehensive brachial artery analysis. Conversely, previous studies in ICU patients demonstrate that accurate brachial artery blood-velocity measurements are easily learned by clinicians with minimal clinical experience [57]. Because our study demonstrates that HV is the brachial artery reactivity parameter that predicts outcomes, future studies can focus exclusively on this measurement, eliminating the need for vessel diameter measurements and specialized expertise. Importantly, previous studies demonstrate that Doppler blood velocity is tightly correlated with plethysmographic blood flow, even over a wide range of arterial flow rates [58,59]. It is therefore likely that our HV measurements reflect the physiological mechanisms ascribed to RH and determined by plethysmography. The use of point-of-care, portable ultrasound in the intensive care unit has grown dramatically [60], so the required equipment for HV measurement already exists in many ICUs, avoiding the additional equipment costs. Finally, HV has recently been measured in large cohort studies to quantify RH and to estimate microvascular function [12-15]. These studies demonstrate that HV is an independent predictor of inflammatory markers, cardiovascular risk factors, and adverse events. For these reasons, HV is an attractive method for measuring RH and assessing microvascular function in future critical care studies.

### FMD in sepsis: comparison with previous studies and measurement challenges

Brachial artery FMD was independently associated with sepsis after controlling for all covariables in subjects younger than 60 years, but not in older individuals. Preexisting age-related endothelial dysfunction or loss of arterial compliance, or both, may explain these findings [61]. Contrary to our original hypothesis, we found no associations between FMD and hospital mortality or severity of illness.

Our findings contrast with those of Vaudo *et al. *[31]. These investigators found that sepsis patients with lower FMD at hospital admission experienced worsening severity of illness (SOFA score) over time. Differences in the patient samples probably account for these conflicting findings. Vaudo *et al. *selected patients with Gram-negative sepsis and did not include patients with preexisting diabetes mellitus, hypertension, smoking, hyperlipidemia, or obesity. In addition, their patients were 41 ± 8 years of age and had no organ dysfunction at enrollment. In contrast, we included unselected consecutive patients with severe sepsis who were older, had greater comorbidity, and had greater severity of illness than did those of Vaudo *et al. *These characteristics probably blunted FMD in our patients, decreasing the measurement signal, and making potential relations between FMD and severity of illness or mortality difficult to detect. Indeed, our FMD results (Table 3) are much lower than those reported by Vaudo *et al. *(8.7 ± 3.6% in sepsis patients and 9.9 ± 1.1% in controls). Although small methodologic differences existed between our study (200 mm Hg cuff inflation for 5 minutes) and Vaudo *et al. *(230 to 250 mm Hg cuff inflation for 4 minutes), it seems unlikely that they contributed substantially to our discrepant findings.

FMD predominantly reflects conduit artery endothelial NO production, although it can be influenced by sympathetic activation [26,27,62]. We found no relation between lower FMD and adverse outcome. It is tempting to conclude that impairments in endothelial function/NO production are not associated with adverse outcomes in patients with severe sepsis. However, the analysis of intraobserver variability suggests the possibility that our FMD measurements were simply unable to detect greater endothelial dysfunction in nonsurvivors. Although measurement of brachial artery diameter was highly repeatable, the FMD measurement error was substantial. This is explained by difficulty quantifying the very small ischemia-induced change in vessel diameter. Importantly, the magnitude, dispersion, and intraobserver variability of our FMD measurements are comparable to those observed in relatively healthy people of similar age in the Framingham cohort studies [63].

We induced stagnant ischemia with the arterial occlusion cuff placed below the antecubital fossa. Upper-arm cuff placement produces a greater FMD magnitude, so it could reduce measurement error [26]. However, the upper-arm occlusion technique is not currently recommended for assessment of conduit artery endothelial function because it may also reflect several additional physiological mechanisms in addtion to endothelial NO production [32]. A recent investigation of an alternative measure of endothelium-dependent vasodilation (change in aortic augmentation index after salbutamol inhalation) demonstrated a strong association with critical illness mortality [64]. This supports the possibility that our FMD measurements were not sufficiently sensitive or precise to detect an association between impaired endothelial function and mortality.

### Relation between HV and FMD in sepsis

To our knowledge, this is the first study comparing the predictive value of FMD and RH in human sepsis. We found that HV and FMD were only weakly correlated, consistent with previous studies [41] and supporting the concept that these two indices of brachial artery reactivity are mediated by different mechanisms [17]. These two indices of brachial artery reactivity were compared in the Framingham Offspring Study [15], showing significant independent relations between inflammatory markers and HV, but not between inflammatory markers and FMD. The authors postulated that inflammation predominantly impairs microvascular function (measured with HV) instead of conduit artery endothelial function (measured with FMD). This conclusion resonates with our findings that HV alone is a powerful predictor of outcomes in the inflammatory milieu of severe sepsis. Again, the negative FMD findings could instead be explained by imprecise quantification of the small change in brachial artery diameter.

### Strengths and limitations

The methodologic strengths of our study are that it included a large, consecutive sample of severe sepsis patients, measurements were made by using a standardized method with trained sonographers, and measurements were analyzed and quantified by a single sonographer blinded to patient outcomes.

Our study has a number of limitations. First, the observational design precludes establishment of a causal relation between low HV and adverse outcome. Proof of causality will require clinical trials testing interventions designed to improve both microvascular function and clinical outcomes. We hope our study will contribute to the rationale for such trials.

Another limitation is that we measured HV and FMD at only one time point during the acute illness, and measurements were often delayed because vascular-access devices were present in the target upper extremity, or sonographers were not immediately available. As shown in Table 2 although most patients initially presented with septic shock, most had recovered normal blood pressure at the time of brachial artery reactivity measurement. It is unknown whether earlier measurements, or serial measurements, would predict clinical outcomes more accurately. That said, it is striking that HV remains impaired and predicts mortality even when systemic blood pressure is normal, suggesting that microvascular dysfunction is pathologically important and persists even after global hemodynamics have stabilized. Our stratified analyses (Table 4) showing that HV remains lower in nonsurvivors, independent of blood pressure or vasopressor use, supports this conclusion as well.

We also must emphasize that HV and FMD measurements were performed by expert sonographers to ensure optimal precision. Therefore, our study is unable to address the feasibility of making similar measurements without registered sonographers. Previous literature provides optimism that accurate HV measurements can be made by other clinicians and investigators [57], but this requires confirmation. Despite this technical expertise, we found substantial intraobserver variability in FMD, as noted earlier. In this context, it is important to note that we manually measured brachial artery boundaries, and we limited our postdeflation 2-D imaging to 30 to 90 seconds after cuff deflation, consistent with original studies and guidelines [26,39,41]. However, more recent guidelines recommend the use of automated edge-detection software to reduce intraobserver variation, and extension of postdeflation 2-D imaging for 3 minutes to identify some patients with delayed peak FMD [32]. These factors together increase the chance of a type II statistical error when we conclude that no association exists between FMD and mortality in severe sepsis.

Another limitation is that we did not measure blood or urine indices of inflammation, oxidative stress, or nitric oxide metabolism at the time of the brachial artery measurements in the severe sepsis cohort, so we are unable to analyze relations between these processes. We also did not routinely measure global hemodynamic variables (for example, central venous pressure or cardiac output), so we are unable to assess their relation with brachial reactivity.

Finally, it is likely that a number of unmeasured differences existed between our relatively healthy control group and the severe sepsis cohort (for example, other comorbidities, sedation, and analgesia). It is therefore possible that such unmeasured covariables confounded the association we observed between brachial artery reactivity and severe sepsis. In a similar way, we cannot determine from this study whether reduced HV is associated with poor outcomes in severe sepsis patients exclusively, or whether it is a more widely applicable prognostic indicator in other critically ill patients.

## Conclusions

In our severe sepsis cohort, both brachial artery FMD and HV were impaired in severe sepsis patients compared with control subjects. However, only HV was a sensitive and specific independent predictor of hospital mortality that was also associated with severity of illness. Our findings are important because they confirm that microvascular dysfunction, assessed by noninvasively measuring HV with commonly available technology, predicts clinical outcomes even after adjustment for important potential confounding variables. We hope this conclusion will encourage the search for novel therapeutic strategies targeting microvascular dysfunction in severe sepsis. Moreover, in clinical trials of these new treatment strategies, our results indicate that brachial artery HV measurements may facilitate enrollment of appropriate patients and assessment of physiologic efficacy. On a cautionary note, if such studies use nonsonographers for HV measurement, the repeatability and precision of their measurements should be validated.

## Key messages

• Impairments in both brachial artery hyperemic velocity and flow-mediated dilation were observed in patients with severe sepsis.

• Impaired hyperemic velocity, but not flow-mediated dilation, predicted hospital mortality with good sensitivity and specificity.

• The association between impaired hyperemic velocity and severe sepsis mortality was independent of blood pressure, vasopressor infusions, age, and multiple comorbid conditions.

• Brachial artery hyperemic velocity may be a clinically useful prognostic tool in severe sepsis.

• Brachial artery hyperemic velocity may be useful in clinical trials targeting microvascular dysfunction in severe sepsis by guiding subject enrollment and measuring physiologic efficacy.

## Abbreviations

AUC: area under the curve; CI: confidence interval; FMD: flow-mediated dilation; HV: hyperemic velocity; ICU: intensive care unit; IQR: interquartile range; NO: nitric oxide; OR: odds ratio; r: brachial artery radius; RH: reactive hyperemia; rho: Spearman rank correlation coefficient; ROC: receiver-operator characteristic; SD: standard deviation; VTI: velocity-time integral.

## Competing interests

The authors declare that they have no competing interests.

## Authors' contributions

OW participated in the statistical analysis and interpretation of the data and drafted the manuscript. MMM, MSG, CMM, DCD, KPD, MJA, and BTG participated in the implementation and conduct of the study. SDS, MWF, and XC participated in the conception and design of the study. APP conceived and designed the study, participated in the implementation and conduct of the study, performed the statistical analyses, interpreted the data, and drafted and finalized the manuscript. All authors read and approved the final manuscript for publication.

## Supplementary Material

Additional file 1 **Online Data Supplement**. Contains tables showing results of stratified and multivariable statistical analyses and figures illustrating intraobserver variability of measurements. Click here for file
