# Supplementary material for: Brachial artery reactivity in patients with severe sepsis: an observational study
Source: Crit Care. 2012 Mar 5;16(2):R38. doi: 10.1186/cc11223 (PMC3568781; doi:10.1186/cc11223)
Supplement: Additional file 1 — Online Data Supplement. Contains tables showing results of stratified and multivariable statistical analyses and figures illustrating intraobserver variability of measurements. [file cc11223-S1.DOC]

**Online Data Supplement**

**Brachial Artery Reactivity in Patients with Severe Sepsis**

Orren Wexler, M.D., Mary Anne M. Morgan, M.D., Michael S. Gough, M.D., Sherry Steinmetz, Cynthia M. Mack, R.N., Denise C. Darling, R.R.T., Kathleen P. Doolin, R.N., Michael J. Apostolakos, M.D., Brian T. Graves, R.N., Mark W. Frampton, M.D.,

Xucai Chen, Ph.D., Anthony P. Pietropaoli, M.D., M.P.H.

**SUPPLEMENTAL TABLES**

Table E1. Flow-mediated dilation (%) in control vs. severe sepsis subjects: stratified analysis *^a^*

| **Covariate** |  |  | **Controls** |  |  | **Severe Sepsis** |  |  | **p *^b^*** |  |  | **Test for interaction *^c^*** |  |
| --- | --- | --- | --- | --- | --- | --- | --- | --- | --- | --- | --- | --- | --- |
| **Age** | |  | | |  | | |  | | |  | | |
| ≤ 60 | | 4.82 (3.76 – 8.33), n=30 | | | 2.65 (0.91 – 4.13), n=45 | | | < 0.0001 | | | p < 0.02 | | |
| > 60 | | 3.56 (1.80 – 5.92), n=22 | | | 2.80 (0.77 – 5.31), n=50 | | | 0.34 | | |  | | |
| **Gender** | |  | | |  | | |  | | |  | | |
| Men | | 3.42 (2.65 – 6.67), n=26 | | | 2.63 (0.91 – 4.52), n=49 | | | 0.04 | | | n/a | | |
| Women | | 4.82 (3.77 – 7.06), n=26 | | | 2.96 (0.80 – 5.06), n=46 | | | 0.001 | | |  | | |
| **Hypertension** | |  | | |  | | |  | | |  | | |
| Yes | | 6.25 (3.16 – 9.32), n=11 | | | 2.64 (0.74 – 4.86), n=59 | | | 0.004 | | | n/a | | |
| No | | 3.88 (2.92 – 6.67), n=41 | | | 2.74 (1.25 – 4.11), n=36 | | | 0.006 | | |  | | |
| **Diabetes Mellitus** | |  | | |  | | |  | | |  | | |
| Yes | | 5.17 (2.99 – 7.06), n=7 | | | 2.16 (0.68 – 4.84), n=27 | | | 0.04 | | | n/a | | |
| No | | 4.08 (3.12 – 6.67), n=45 | | | 2.97 (1.06 – 4.68), n=68 | | | 0.002 | | |  | | |
| **Hyperlipidemia** | |  | | |  | | |  | | |  | | |
| Yes | | 3.82 (3.26 – 7.76), n=16 | | | 3.00 (1.84 – 4.79), n=34 | | | 0.082 | | | p > 0.50 | | |
| No | | 4.22 (3.02 – 6.67), n=36 | | | 2.56 (0.77 – 4.76), n=61 | | | < 0.001 | | |  | | |
| **Active smoking** | |  | | |  | | |  | | |  | | |
| Yes | | 9.76, n=1 | | | 2.65 (1.36 – 5.88), n=23 | | | 0.097 | | | unknown*^d^* | | |
| No | | 4.08 (2.99 – 6.67), n=51 | | | 2.74 (0.76 – 4.29), n=72 | | | < 0.001 | | |  | | |
| **Coronary artery disease** | |  | | |  | | |  | | |  | | |
| Yes | | 7.02 (1.35 – 10), n=3 | | | 2.65 (2.1 – 6.76), n=17 | | | 0.186 | | | p > 0.50 | | |
| No | | 4.08 (3.12 – 6.67), n=49 | | | 2.74 (0.8 – 4.84), n=78 | | | < 0.001 | | |  | | |
| **Blood pressure** | |  | | |  | | |  | | |  | | |
| MAP ≤ 90 | | 4.22 (3.36 – 7.04), n=24 | | | 2.64 (0.79 – 4.85), n=72 | | | 0.001 | | | n/a | | |
| MAP > 90 | | 3.83 (2.49 – 6.67), n=28 | | | 2.96 (0.91 – 4.58), n=23 | | | 0.03 | | |  | | |
| **Charlson index** | |  | | |  | | |  | | |  | | |
| < 1 | | 4.30 (3.60 – 6.67), n=37 | | | 4.08 (1.14 – 5.68), n=11 | | | 0.128 | | | p > 0.5 | | |
| ≥ 1 | | 3.52 (2.24 – 7.06), n=15 | | | 2.63 (0.78 – 4.67), n=84 | | | 0.068 | | |  | | |

*Definition of abbreviations*: MAP = mean arterial pressure; n/a = not applicable; Charlson index = Charlson comorbidity index [1]

^a^ Continuous variables were dichotomized according to the median value of all subjects for this analysis

^b^ p-value refers to the comparison of FMD between survivors and non-survivors within the specified subgroup.

*^c^* The test for interaction [2] was conducted when stratified analysis showed that one of the subgroups within a specific covariable was not statistically significant, suggesting heterogeneity in the relationship between FMD and mortality within strata of that covariable. The row is labeled “n/a” if this condition was not met.

*^d^*The test for interaction could not be completed because there was only one smoking control subject so the standard error of FMD in smoking control subjects could not be defined.

Table E2. Hyperemic velocity (cm/cardiac cycle) in control vs. severe sepsis subjects: stratified analysis *^a^*

| **Covariable** |  |  | **Controls** |  |  | **Severe Sepsis** |  |  | **p *^b^*** |  |  | **Test for interaction *^c^*** |  |
| --- | --- | --- | --- | --- | --- | --- | --- | --- | --- | --- | --- | --- | --- |
| **Age** | |  | | |  | | |  | | |  | | |
| ≤ 60 | | 70 (58 – 85), n=30 | | | 37 (28 – 51), n=45 | | | < 0.001 | | | n/a | | |
| > 60 | | 62 (42 – 78), n=22 | | | 34 (25 – 46), n=50 | | | < 0.001 | | |
| **Gender** | |  | | |  | | |  | | |  | | |
| Men | | 59 (42 – 80, n=26 | | | 34 (26 – 46), n=49 | | | < 0.001 | | | n/a | | |
| Women | | 71 (61 – 82), n=26 | | | 36 (25 – 51), n=46 | | | < 0.001 | | |
| **Hypertension** | |  | | |  | | |  | | |  | | |
| Yes | | 73 (53 – 82), n=11 | | | 32 (25 – 46), n=59 | | | < 0.001 | | | n/a | | |
| No | | 62 (52 – 80), n=41 | | | 38 (30 – 50), n=36 | | | < 0.001 | | |
| **Diabetes Mellitus** | |  | | |  | | |  | | |  | | |
| Yes | | 62 (51 – 82), n=7 | | | 26 (21 – 51), n=27 | | | < 0.001 | | | n/a | | |
| No | | 64 (53 – 80), n=45 | | | 37 (28 – 47), n=68 | | | < 0.001 | | |
| **Hyperlipidemia** | |  | | |  | | |  | | |  | | |
| Yes | | 60 (50 – 83), n=16 | | | 34 (22 – 44), n=34 | | | < 0.001 | | | n/a | | |
| No | | 68 (52 – 80), n=36 | | | 36 (27 – 49), n=61 | | | < 0.001 | | |  | | |
| **Active smoking** | |  | | |  | | |  | | |  | | |
| Yes | | 58, n=1 | | | 31 (26 – 52), n=23 | | | 0.220 | | | Unknown*^d^* | | |
| No | | 64 (52 – 82), n=51 | | | 35 (25 – 46), n=72 | | | < 0.001 | | |  | | |
| **Coronary artery disease** | |  | | |  | | |  | | |  | | |
| Yes | | 53 (38 – 82), n=3 | | | 34 (26 – 44), n=17 | | | 0.064 | | | p < 0.50 | | |
| No | | 64 (53 – 80), n=49 | | | 35 (25 – 49), n=78 | | | < 0.001 | | |  | | |
| **Blood pressure** | |  | | |  | | |  | | |  | | |
| MAP < 85 | | 60 (53 – 67), n=13 | | | 34 (25 – 44), n=58 | | | < 0.001 | | | n/a | | |
| MAP ≥ 85 | | 70 (52 – 82), n=39 | | | 38 (28 – 51), n=37 | | | < 0.001 | | |
| **Charlson index** | |  | | |  | | |  | | |  | | |
| < 1 | | 69 (57 – 80), n=37 | | | 41 (27 – 51), n=11 | | | < 0.001 | | | n/a | | |
| ≥ 1 | | 58 (42 – 82), n=15 | | | 34 (25 – 47), n=84 | | | < 0.001 | | |

*Definition of abbreviations*: MAP = mean arterial pressure; n/a = not applicable; Charlson index = Charlson comorbidity index [1]

^a^ Continuous variables were dichotomized according to the median value of all subjects for this analysis

^b^ p-value refers to the comparison of HV between survivors and non-survivors within the specified subgroup.

*^c^* The test for interaction [2] was conducted when stratified analysis showed that one of the subgroups within a specific covariable was not statistically significant, suggesting heterogeneity in the relationship between HV and mortality within strata of that covariable. The row is labeled “n/a” if this condition was not met.

*^d^*The test for interaction could not be completed because there was only one smoking control subject so the standard error of HV in smoking control subjects could not be defined.

Table E3. Multivariable analyses evaluating association between FMD and severe sepsis

|  | | **Association between FMD and severe sepsis in subjects ≤ 60 years** | | | |  |
| --- | --- | --- | --- | --- | --- | --- |
|  | **Dependent variable** | | **Independent variables** | **Regression coefficient (95% CI)** | ***p* value** | |
|  | Severe sepsis | | FMD | - 0.498 (- 0.854, - 0.142) | 0.006 | |
|  |  | | Female gender | 0.337 (- 1.316, 1.990) | 0.69 | |
|  |  | | history of hypertension | 1.908 (- 0.341, 4.158) | 0.10 | |
|  |  | | mean arterial blood pressure | - 0.116 (- 0.199, - 0.034) | 0.006 | |
|  |  | | Charlson index | 1.322 (0.434, 2.210) | 0.004 | |
|  |  | | constant | 10.714 (3.131, 18.296) | 0.006 | |
|  | | **Association between FMD and severe sepsis in subjects > 60 years** | | | |  |
|  | **Dependent variable** | | **Independent variables** | **Regression coefficient (95% CI)** | ***p* value** | |
|  | Severe sepsis | | FMD | - 0.064 (- 0.230, 0.102) | 0.45 | |
|  |  | | Female gender | 2.018 (0.377, 3.659) | 0.02 | |
|  |  | | history of hypertension | 1.953 (0.426, 3.480) | 0.01 | |
|  |  | | mean arterial blood pressure | - 0.076 (- 0.139, - 0.014) | 0.02 | |
|  |  | | Charlson index | 0.454 (0.113, 0.794) | 0.01 | |
|  |  | | constant | 4.642 (-0.782, 10.066) | 0.09 | |

*Definition of abbreviations:* FMD = flow-mediated dilation (%); CI = confidence interval; Charlson index = Charlson comorbidity index [1]

Table E4. Multivariable analyses evaluating role of hyperemic velocity in predicting severe sepsis

|  | |  | | | |  |
| --- | --- | --- | --- | --- | --- | --- |
|  | **Dependent variable** | | **Independent variables** | **Odds ratio *^a^* (95% CI)** | ***p* value** | |
|  | Severe sepsis | | HV | 1.05 (1.02, 1.08) | 0.001 | |
|  |  | | age | 0.94 (0.90, 0.98) | 0.007 | |
|  |  | | Female gender | 2.63 (0.87 – 7.89) | 0.08 | |
|  |  | | history of hypertension | 7.77 (2.13, 28.39) | 0.002 | |
|  |  | | mean arterial blood pressure | 0.94 (0.91, 0.98) | 0.006 | |
|  |  | | Charlson index | 1.74 (1.21, 2.50) | 0.003 | |

***^a^***Odds ratios refer to the change in likelihood of severe sepsis per 1 cm/ cardiac cycle decrease in HV, per 1 year increase in age, for females vs. males, for those with vs. without a medical history of hypertension, per 1 mm Hg increase in blood pressure, and per one point increase in Charslon index.

Table E5. Multivariable analysis: association between hyperemic velocity and hospital mortality

|  | **Dependent variable** | **Independent variables *^a^*** | **Odds ratio (95% CI) *^b^*** | ***p* value** |
| --- | --- | --- | --- | --- |
|  | Hospital mortality | HV | 1.11 (1.04, 1.19) | 0.003 |
|  |  | Age | 1.06 (1.01, 1.11) | 0.021 |
|  |  | Diabetes mellitus | 3.32 (0.86 – 12.92) | 0.083 |

*Definition of abbreviations:* HV = hyperemic velocity (cm/ cardiac cycle); CI = confidence interval

*^a^*The initial model also included medical history of hypertension, hyperlipidemia, smoking, coronary artery disease, gender, mean arterial blood pressure and vasopressor use at the time of brachial artery reactivity measurements, and Charlson comorbidity index [1]. These covariables were removed during model building without significant deterioration in model fit, as described in Methods. ***^b^*** Odds ratios refer to the change in likelihood of hospital mortality per 1 cm/ cardiac cycle decrease in HV, per 1 year increase in age, and for patients with vs. without a medical history of diabetes mellitus.

**SUPPLEMENTAL FIGURES**Figure E1. Scatterplot of 1^st^ measurement (x axis) and 2^nd^ measurement (y axis) for hyperemic velocity (A), flow-mediated dilation (B), baseline brachial artery diameter (C) and hyperemic brachial artery diameter (D). The dashed lines are the lines of identity that signify perfect agreement between measurements.


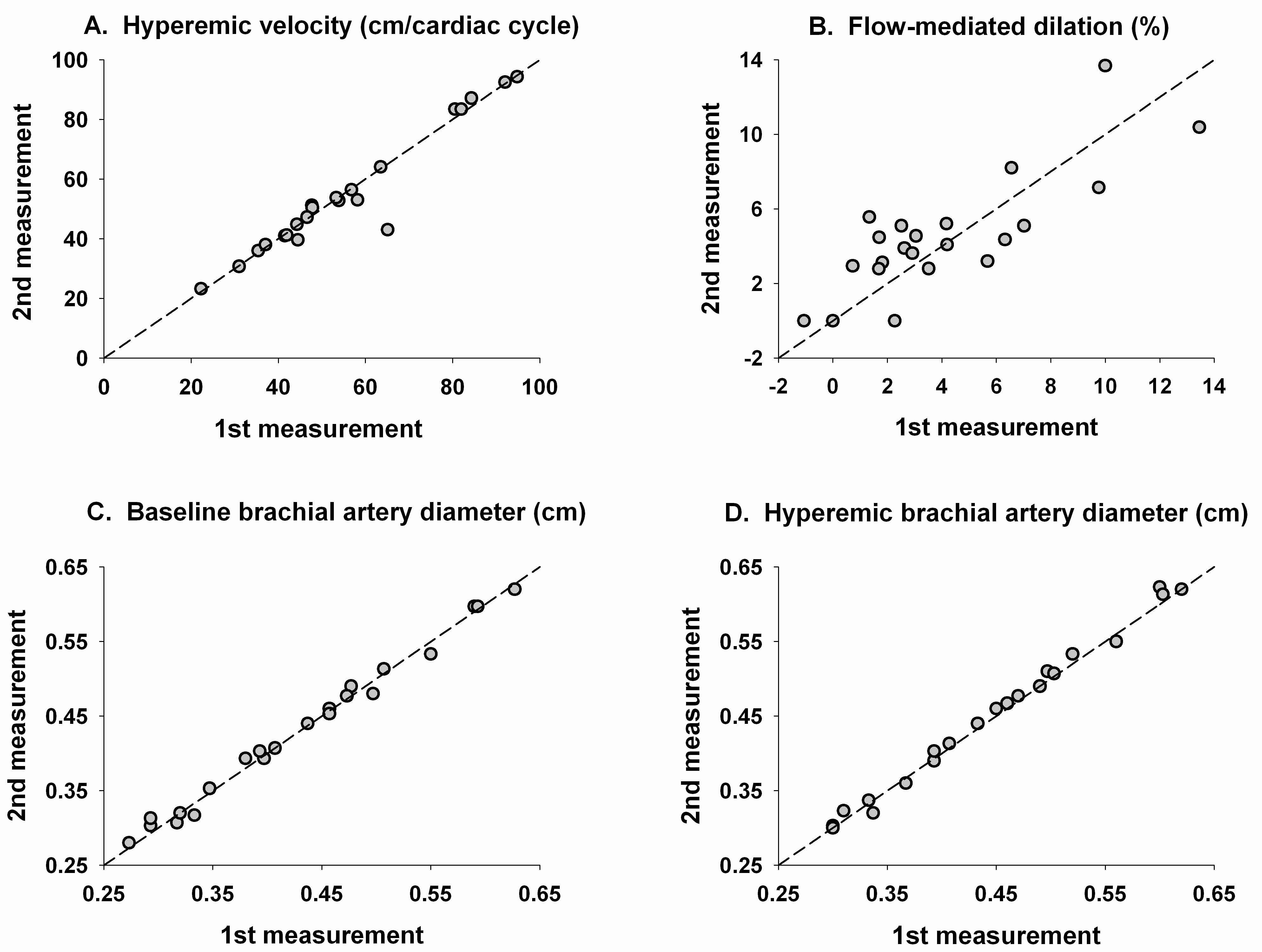


Figure E2. Bland – Altman plots [3]for assessing measurement agreement for hyperemic velocity (A) and flow-mediated dilation (B). Each of the differences between paired measurements is plotted in the y axis and the average value for each measurement pair is plotted on the y-axis. The dashed line represents the mean difference between paired measurements, and the dotted/ dashed lines represent the mean difference between paired measurements ± 2 standard deviations. The plots illustrate that although the average measurement error (dashed line) is close to zero in both panels, the range of measurement error for FMD is high relative to the magnitude of the measurements themselves (represented on the x axis). This is not so for HV, except for the one obvious outlier (included in the intra-observer variability analyses).


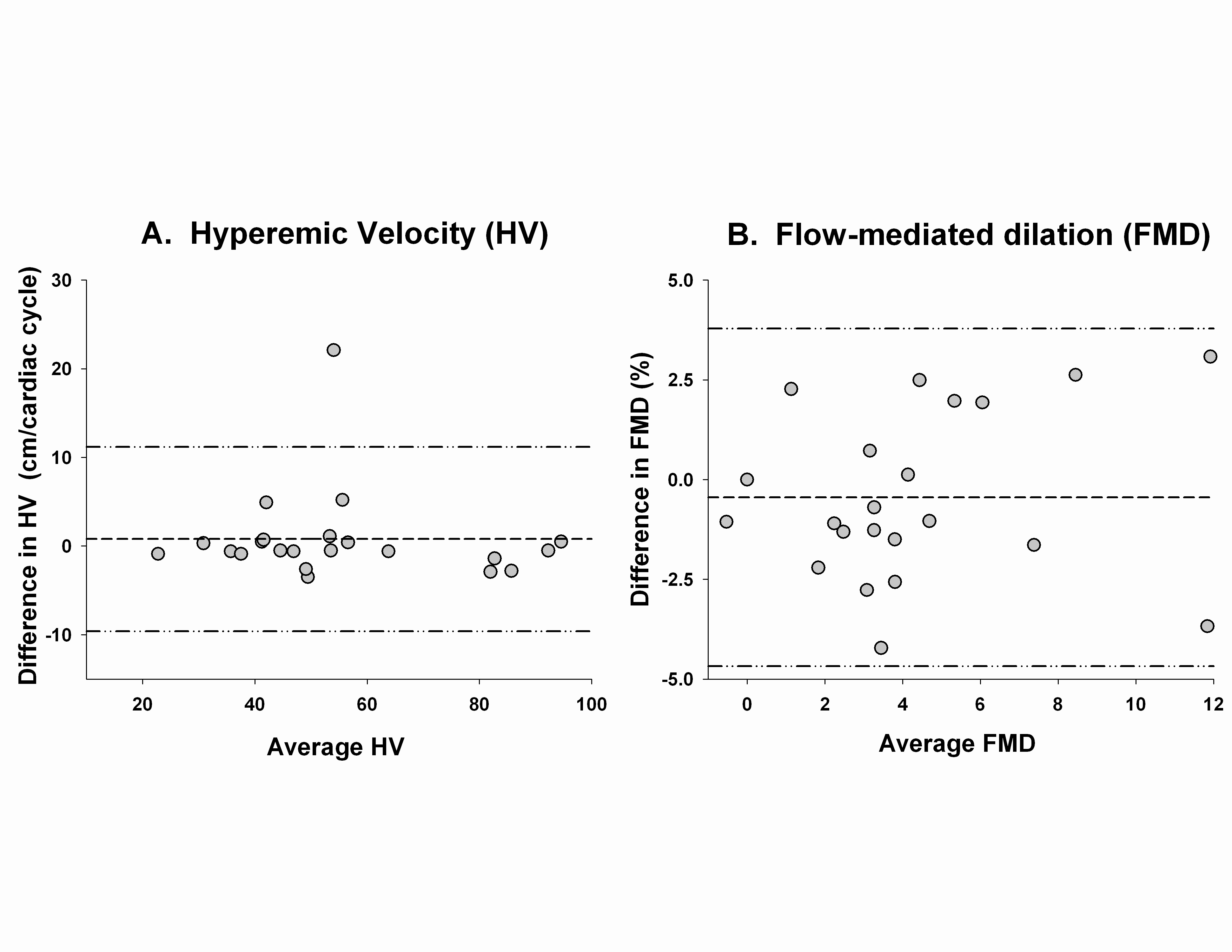


Figure E3. Bland – Altman plots [3] for assessing measurement agreement for baseline (A) and hyperemic (B) brachial artery diameter. Each of the differences between paired measurements is plotted in the y axis and the average value for each measurement pair is plotted on the y-axis. The dashed line represents the mean difference between paired measurements, and the dotted/ dashed lines represent the mean difference between paired measurements ± 2 standard deviations. The plots illustrate that the average measurement error (dashed line) is close to zero and the range of measurement error (represented on the y axis) is small relative to the magnitude of the measurements themselves (represented on the x axis).


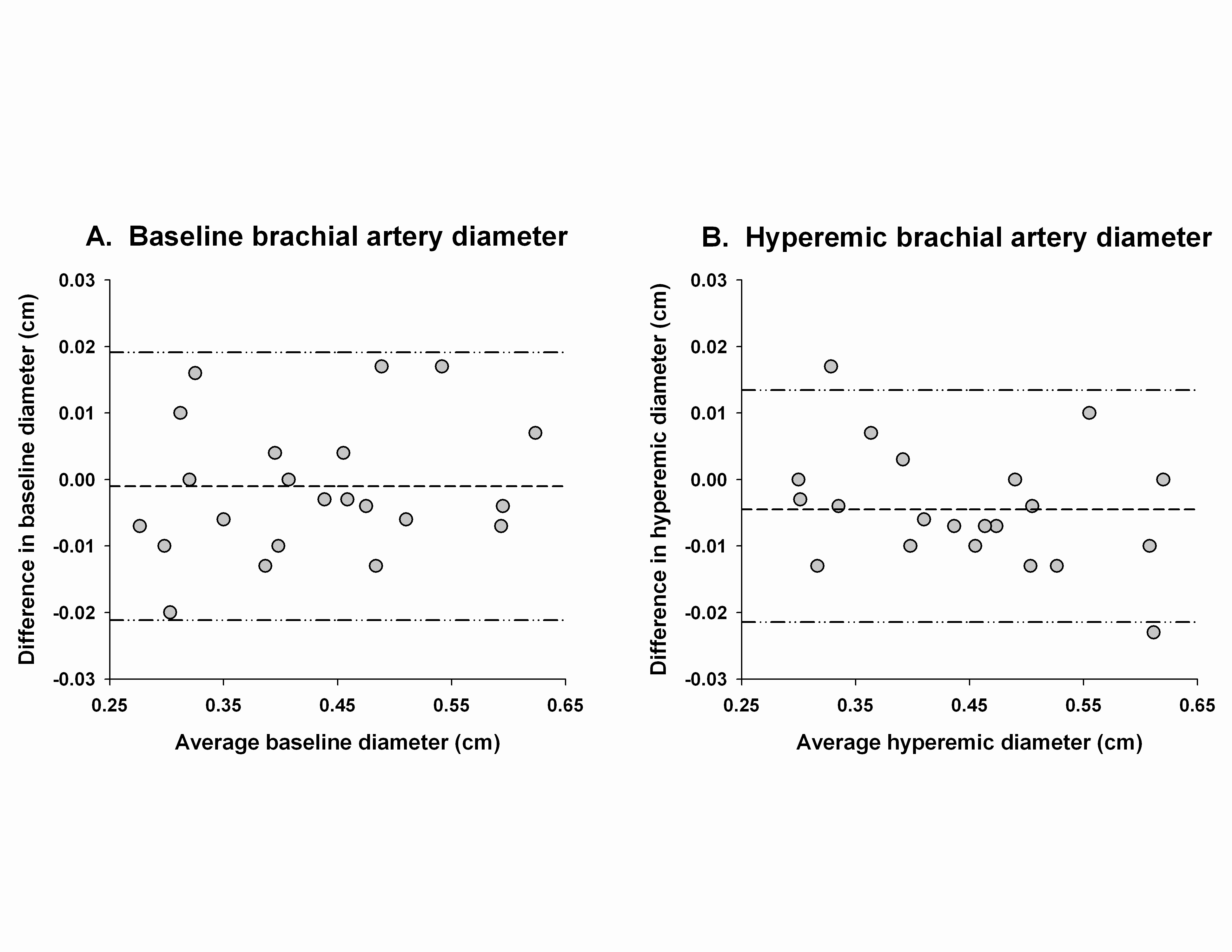


**SUPPLEMENTAL REFERENCES**

1. Charlson ME, Pompei P, Ales KL, MacKenzie CR: **A new method of classifying prognostic comorbidity in longitudinal studies: development and validation**. *J Chronic Dis* 1987, **40**(5):373-383.

2. Matthews JN, Altman DG: **Interaction 3: How to examine heterogeneity**. *BMJ* 1996, **313**(7061):862.

3. Bland JM, Altman DG: **Statistical methods for assessing agreement between two methods of clinical measurement.** *Lancet* 1986, **1**(8476):307-310.
